# Biosynthesis and Molecular Genetics of Polyketides in Marine Dinoflagellates

**DOI:** 10.3390/md8041011

**Published:** 2010-03-31

**Authors:** Ralf Kellmann, Anke Stüken, Russell J. S. Orr, Helene M. Svendsen, Kjetill S. Jakobsen

**Affiliations:** 1 University of Bergen, Department of Molecular Biology, 5020 Bergen, Norway; E-Mail: Helene.Svendsen@student.uib.no; 2 University of Oslo, Department of Biology, Centre for Ecological and Evolutionary Synthesis (CEES), 0316 Oslo, Norway; E-Mails: anke.stuken@web.de (A.S.); k.s.jakobsen@bio.uio.no (K.S.J.); 3 University of Oslo, Department of Biology, Microbial Evolution Research Group (MERG), 0316 Oslo, Norway; E-Mail: russell.orr@bio.uio.no

**Keywords:** polyketides, polyether toxins, alkaloids, molecular genetics, genomics, biosynthesis

## Abstract

Marine dinoflagellates are the single most important group of algae that produce toxins, which have a global impact on human activities. The toxins are chemically diverse, and include macrolides, cyclic polyethers, spirolides and purine alkaloids. Whereas there is a multitude of studies describing the pharmacology of these toxins, there is limited or no knowledge regarding the biochemistry and molecular genetics involved in their biosynthesis. Recently, however, exciting advances have been made. Expressed sequence tag sequencing studies have revealed important insights into the transcriptomes of dinoflagellates, whereas other studies have implicated polyketide synthase genes in the biosynthesis of cyclic polyether toxins, and the molecular genetic basis for the biosynthesis of paralytic shellfish toxins has been elucidated in cyanobacteria. This review summarises the recent progress that has been made regarding the unusual genomes of dinoflagellates, the biosynthesis and molecular genetics of dinoflagellate toxins. In addition, the evolution of these metabolic pathways will be discussed, and an outlook for future research and possible applications is provided.

## 1. Introduction

Dinoflagellates are unicellular biflagellates that belong to the ancient eukaryotic lineage Alveolata, which also includes two other clades, the ciliates and apicomplexan parasites. Approximately 2000 species of dinoflagellates exist, and most of these are marine species [1]. Dinoflagellates are most commonly known for their capacity to form harmful algal blooms (HABs). Although the number of harmful dinoflagellate species is small (less than 100 species) [2,3], they negatively impact human activities and local ecosystems on a global scale, due to their world-wide distribution, their capacity to mass proliferate, and to produce toxins that accumulate through marine food webs [4]. All but one of the classical seafood poisoning syndromes, paralytic- (PSP), diarrhetic- (DSP), neurotoxic- (NSP), azaspiracid shellfish poisoning (AZP), and ciguatera fish poisoning (CFP), are caused by dinoflagellate toxins. The only exception is amnesic shellfish poisoning (ASP), which is caused by the diatomean toxin, domoic acid [4]. In addition to the classic seafood toxins, dinoflagellates may produce a range of other biologically active compounds, including neurotoxins, cytotoxins, antibiotics, and immunosuppressants [5–7]. Some of these metabolites have been difficult to identify, such as the ichtyotoxins produced by strains of the *Pfiesteria* species complex [8–10]. To date, approximately 45 metabolite families are known from dinoflagellates, some with therapeutic potential [6,11]. Although this is a small number in comparison to a total of nearly 20000 bioactive compounds discovered over the past 40 years [12], dinoflagellate metabolites are unique in terms of their size, structural complexity, and potency in some cases [6]. All the metabolites described so far from dinoflagellates are of polyketide origin [6,11,13].

Polyketides are produced by successive condensations of carboxylic acid extender units to a growing acyl chain in a similar way as in fatty acid biosynthesis. Structural diversity is achieved by several means. Alternative extender units, such as acetate, malonate, propionate, butyrate, and glycolate may be used. In addition, one or several of the post-condensation reactions, β-ketoacyl reduction, a dehydration step, and an enoyl reduction, which follow each acyl chain elongation cycle in fatty acid biosynthesis, may be omitted [14]. Finally, many polyketide biosynthesis pathways have integrated enzymes from other metabolic pathways to add new functionalities to the polyketide chain [15,16]. Polyketide synthesis may for instance be combined with non-ribosomal peptide synthesis, where carboxylic and amino acid extender units are sequentially added to a growing acyl or peptidyl chain. The enzymes carrying out polyketide synthesis, polyketide synthases (PKSs) have been classified into three types, depending on their domain organisation, and whether these enzymes were iterative [17]. Many PKS enzymes have recently been identified, however, which do not conform to this classification, casting doubts regarding its validity [18].

The chemical structures of polyketides from dinoflagellates suggest that they are produced by type I polyketide synthases (PKSs), in some cases with the involvement of a non-ribosomal peptide synthetase (NRPSs) [11]. Type I PKSs and NRPSs usually consist of large, non-iterative, multi-domain enzymes [19]. Modular type I PKSs and NRPSs form megasynthetases that generally follow a colinearity rule [20], where one module extends a growing acyl or peptidyl chain by one particular unit. The colinearity of type I PKS, and NRPS enzymes enables the prediction of the chemical structure of their metabolite products [20].

A wealth of knowledge is available on the biosynthesis and enzymology of polyketides from terrestrial, or freshwater organisms [21–23], but little is known regarding polyketides from dinoflagellates. Several factors have hampered studies into the biochemistry and molecular genetics of polyketides from dinoflagellates. Many species are difficult to culture and they can rarely be maintained in an axenic state [11]. They do not readily take up isotope-labeled precursors, because of their autotrophic nature, and in addition, they provide a number of features at the cellular and molecular genetic level that are not known from other organisms: they have extremely large genomes and lack normal chromosome organization, they exhibit an unusual gene organization and a high proportion of modified bases in DNA; features that will be discussed in the following section. In spite of these difficulties, substantial progress has been made recently, due to improved genomic methods, such as high throughput pyrosequencing. EST (cDNA) sequencing projects have now been performed on nine dinoflagellate species and targeted studies have uncovered the first PKS genes from dinoflagellates. This review will summarize the recent progress with emphasis on the molecular genetics of dinoflagellate polyketides.

## 2. Dinoflagellates Have Peculiar Cellular and Genomic Features and an Evolutionary History of Multiple Endosymbioses

Dinoflagellates are most closely related to ciliates and apicomplexians (malaria parasites) forming the alveolate group. The alveolates, in turn, together with the chromist groups (heterokonts, haptophytes and cryptomonades), have been suggested to form the chromalveolate lineage, a supergroup that is thought to have a common origin through an endosymbiontic event, which led to their red plastid. However, in dinoflagellates a diversity of plastids is found. Although red algal-derived pigments are present in peridinin plastids, which are believed to be the ancestral plastid of this group, the various dinoflagellate plastids are not all of red algal origin. Some plastids are green, and derived from green algae, whereas others are derived from tertiary endosymbiosis from haptophytes [24,25], while others again represent transient kleptoplastids [26,27]. Multigene phylogenies employing hundreds of protein-encoding genes, and sophisticated phylogenetic inference methods have revealed that the idea of chromalveolates originating from a single endosymbiosis event is not necessarily true, and that plastids have been acquired and lost more often than hitherto appreciated [28–30]. Based on such multigene phylogenies, reshuffling of the eukaryotic supergroups with implications for the chromalveolate idea has recently been proposed [28,29,31]. It now appears that recurrent endosymbioses involving plastid replacements, and several rounds of endosymbiontic gene transfer (EGF) are likely to have occurred in many eukaryotic groups, and that the dinoflagellates are a prime example of multiple plastid replacements [32]. Phylogenies of nuclear-encoded genes have displayed plastid gene affinity to several protist groups, such as green-algae, stramenophiles, haptophytes and red algae for the peridinin dinoflagellates [30]. Furthermore, the dinoflagellate species with deviant plastids, such as haptophyte-derived (*Karlodinium veneficum*, *Karenina brevis*), and green algal-derived (*Lepidodinium chlorophorum*) plastids, an even larger phylogenetic spectrum as seen for the peridinin dinoflagellates have been implicated [33–35]. Some of these genes may represent previous (cryptic) endosymbioses, while others may in fact be associated with horizontal gene transfer (HGT). For example, some genes seem to have a clear prokaryotic origin [35,36]. Thus, the dinoflagellate nuclear genome has been shaped by EGF and HGT, and from an evolutionary standpoint displays a stronger flavour of phylogenetic mosaics than most other genomes we know of [30,34–36].

The dinoflagellate nucleus is remarkable in several ways. The chromosomes are condensed throughout the cell cycle, and the DNA is not organized in normal nucleosomes, but tightly packaged around a few, low molecular weight histone-like proteins (see Koumandou *et al.* [37] and references therein). The lack of histones and normal chromosome organization was originally interpreted as dinoflagellates being an intermediate between eukaryotes and prokaryotes, and therefore termed mesokaryotes [38]. Current knowledge clearly undermines this view. Dinoflagellates are true eukaryotes, however, they do have many unusual features. During cell division, the chromosomes are attached to the nuclear membrane, which persists throughout cell division, and a typical spindle apparatus is lacking. The dinoflagellate genome is highly unconventional at all levels – from the genome size, base composition, gene arrangements to the transcriptional level. The dinoflagellate genomes are large–generally much larger than most algal and protist genomes ranging from 1.5 pg up to 250 pg per haploid genome [39], and are therefore magnitudes larger than the human and other vertebrate genomes. Dinoflagellate DNA is unusual in that it contains 5-hydroxymethyluracil (5-meU) replacing as much as 70% of the thymine [40]. In addition, it also contains substantial amounts of other modified bases, such as 5-methyl cytosine, and N6-methyl adenine [41,42]. The biological meaning of 5-MeU is not clear, but it has been suggested that it is associated with a restriction-modification system for discerning between dinoflagellate DNA, and foreign DNA sequences [41]. It may also be the case that the presence of 5-meU is caused by a lack of a DNA repair mechanism for removal of 5-meU accumulating in the genome (5-meU DNA glycosylase activity) [43].

The organization of genes is also peculiar. In the peridinin plastid, the usual plastid genome has been broken down to minicircles with a single gene (or even no gene) [37,44]. Less than 20 genes have altogether been found on minicircles, implying that most of the genes have been lost from the plastid genome, and transferred to the nuclear genome [37]. The mitochondrial genome has also an unusual and fragmented organization [45]. Less is currently known about the nuclear genome of dinoflagellates, but some quite peculiar features are emerging. Many dinoflagellate genes are organized in multiple copies - as tandem repeats [46]. Such genes usually have a low intron density, or even no introns, and many of them are shown to be highly expressed [46]. Some genes seem, however, to be more intron-rich. For example, a PKS (ketoyl reductase domain) has 18 introns [46]. Most of the investigated genes in dinoflagellates possess a unique leader sequence that originates from a spliced leader (SL) trans splicing mechanism, in which a short RNA fragment (*i.e.*, SL; 15–50 nt) from a small non-coding RNA (SL RNA) is spliced to an acceptor site in the 5′ untranslated region of independently transcribed pre-mRNAs [47,48]. It seems that SL trans-splicing is a universal feature of dinoflagellates. However, the trans-splicing phenomenon is also found in a range of organisms, including euglenozoans, nematodes, platyhelmintes, cnirdarians, rotifers ascidians and appendicularia. Notably, the dinoflagellate SL sequence (conserved in all dinoflagellate lineages) shows no similarity to its counterpart in other organisms, and evidence is emerging for multiple and independent origins of the trans-splicing process [49]. The function of this trans-splicing process is unknown in dinoflagellates, but it is not unlikely that at least one function could be to generate translatable monocistronic mRNAs from polycistronic transcripts [50]. It may seem likely that many of the intron-less (or intron-scarce) genes are the result of incorporation of cDNAs into the dinoflagellate genome through a “retro-mechanism” [51]. The total number of genes in the dinoflagellate genome seems to be much higher than in all other (known) eukaryotic groups. Recent calculations based on gene number-genome size regressions of sequenced eukaryotic genomes have predicted as much as 90.000 genes in several dinoflagellate genomes [52]. The proposed retroactivity and horizontal gene transfer (HGT) may contribute to the high number of genes. In addition to trans-splicing, it is evident that many dinoflagellate genes, particularly in the organelles (both mitochondria and plastids), but also nuclear genes, undergo RNA editing. Dinoflagellates show the same base changes as have been shown for other species, but in addition some changes specific for this group [53,54].

The above features and peculiarities of dinoflagellate genomes are of strong relevance for finding, identifying and understanding the structure of genes encoding enzymes involved in toxin and natural product biosynthesis. First of all, the toxin genes in dinoflagellates may be quite differently organized than similar genes in other eukaryotic organisms and bacteria. Editing and trans-splicing may be essential for functionality, and it is unclear, whether or not the various required enzymatic activities (or enzymes) are encoded by tightly linked genes.

## 3. Precursor Incorporation Studies Reveal a Novel Mode of Polyketide Synthesis in Dinoflagellate

### 3.1. Polyether ladder toxins

**Figure 1:**
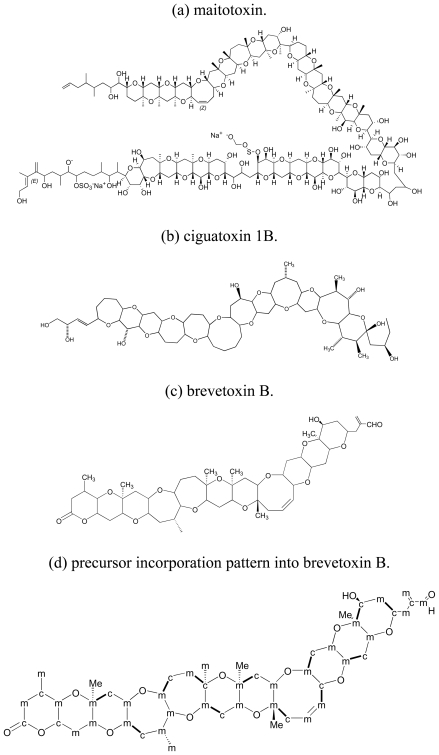

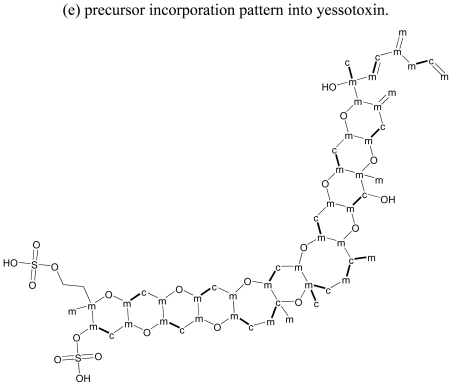
Polyether ladder toxins from dinoflagellates. (**a**) maitotoxin, (**b**) ciguatoxin 1B, (**c**) brevetoxin B [64], (**d**) precursor incorporation pattern into brevetoxin B (**e**) precursor incorporation pattern into yessotoxin [65]. Stable isotope-labeled precursor incorporation patterns are shown for brevetoxin B, and yessotoxin. m: acetate methyl, c: acetate carboxyl, Me: methionine methyl. Bold bonds between c and m indicate intact acetate units.

At least three structural classes of polyether compounds from dinoflagellates can be distinguished: polyether ladders, linear polyethers, and macrolides. The molecular backbone of polyether ladders consists of all *trans*-fused cyclic ether rings (Figure 1), and they include the seafood toxins, ciguatoxins (CTXs), brevetoxins (BTXs), maitotoxin (MTX), and yessotoxins (YTXs).

CTXs and MTX are the causative toxins of ciguatera fish poisoning (CFP). Although the mortality of CFP is relatively low (≈1%), it affects the largest number of humans (50.000 per year) out of all algal-toxin related seafood poisoning syndromes [55]. CTXs are highly potent agonists of voltage-gated sodium channels [56,57]. Twelve isoforms are known, which may cause few to over 30 different neurological symptoms [58]. MTX is the largest, as well as the most potent non-peptide, non-polymeric toxin [6,59]. It activates calcium channels in receptor-mediated processes and thereby affects neurotransmission, hormone release, and phospholipid metabolism [60–62]. Both toxin groups are produced by the tropical epiphytic dinoflagellate, *Gambierdiscus toxicus,* which is often ingested by herbivorous fish on coral reefs. *G. toxicus* is difficult to culture [63] and biosynthetic studies have not been performed on CTX and MTX.

Neurotoxic shellfish poisoning (NSP) is caused by BTXs, which are similar in their chemical structure and pharmacology to CTXs, however they cause milder symptoms. They act on voltage-gated sodium channels, and depolarize membranes of excitable cells [66]. BTXs are produced by strains of *K. brevis* [67,68] and several biosynthetic studies have been performed, as described below.

YTXs have previously been misclassified as diarrhetic (DSP) toxins, but later studies have shown that these toxins have low oral toxicity in mice, and did not inhibit the targets of DSP toxins, protein phosphatases PP2A/PP1 [69]. When YTXs were injected into mice, they were highly toxic, causing multiple organ damage [70]. The mechanism for YTX toxicity remains unknown, however, YTXs have not been implicated in human poisoning so far. Approximately 22 structural analogs of YTXs are known [71], which are produced by various dinoflagellates, including *Protoceratium reticulatum* [72], *Lingulodinium polyedrum* [73] *and Gonyaulax spinifera* [74]. A single biosynthetic study has been performed on one of the YTX analogs, as described below.

### 3.2. Precursor incorporation studies on polyether ladder toxins

**Figure 2:**
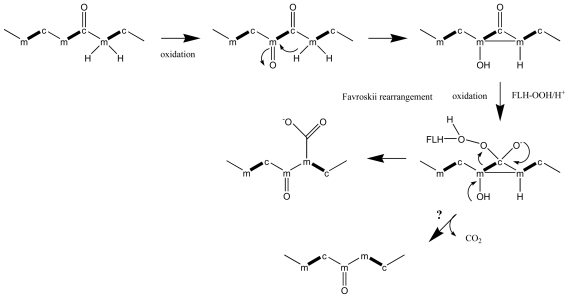
Favorskii-like rearrangement implicated in C1-carbon deletion and acetate-methyl side-chain generation in dinoflagellates. (**a**) C1-carbon deletion mechanism implicated in dinoflagellates. (**b**) Introduction of a methyl side-chain.

The polyether ladders are uniquely found in dinoflagellates and their high structural similarities suggest common biosynthetic mechanisms that are involved in their synthesis [64,68,75,76]. Initial precursor incorporation studies on BTX found a head-to-tail arrangement of acetate units over much of its carbon skeleton, whereas pendent methyl groups were derived from methionine methyl [77]. However, an unusual pattern was observed, where C1 carbons of acetate were frequently missing, and pendant methyl groups were derived from acetate methyl, in addition to *S*-adenosyl methionine (SAM) [64,77,78]. C1-deletions, and pendant methyl groups derived from acetate methyl were subsequently detected in many other polyketides from dinoflagellate [11]. Although this chemistry is unusual, there is precedence for C1-deletions in polyketides from bacteria, such as in ambruticin and enterocin biosynthesis [79,80]. In the case of enterocin, a flavin-dependent monooxygenase, EncM, was demonstrated to catalyse an oxidative Favorskii rearrangement that leads to a pendant carboxyl group, which originated from C1 of acetate (Figure 2) [79].

**Figure 3:**
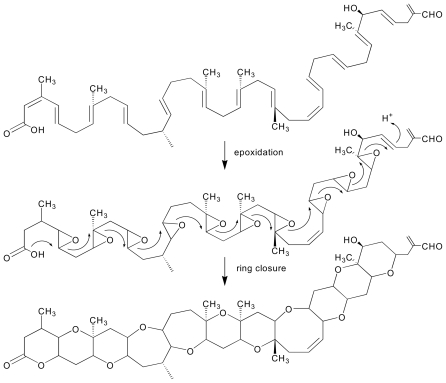
Polyepoxide cascade mechanism involved in the generation of cyclic polyether rings.

The cyclic trans-polyethers were proposed to be formed in a polyepoxide cascade mechanism (Figure 3), which is primed at one end of the molecule, and then propagates in a self-sustaining cascade across the multiple epoxide groups along the molecule as observed for brevisamide [84,85] and monensin [86]. In the case of monensin, two enzymes have been implicated in polyether ring formation, MonCI, a non-heme epoxidase, which may epoxidate three double bonds of monensin precursor, and MonCII, an epoxide hydrolase, which was proposed to open the epoxides with concerted cyclization to produce four polyether rings [86]. A detailed review of the chemistry involved in the cyclization of polyether compounds is reviewed by Vilotijevic and Jamison [87] in this issue of Marine Drugs.

BTXs have a terminal aldehyde group, which is derived from an acetate methyl carbon) Terminal aldehyde groups are generally not found in polyketides. A likely explanation for its origin in BTX has been put forward. The terminal β-keto-group of a polyketide chain is reduced to an alcohol, and subsequently dehydrated to an α,β-unsaturated acid, as part of conventional polyketide synthesis. This double bond may then be subjected to epoxidation, followed by spontaneous decarboxylation, which leaves behind a terminal aldehyde on a polyketide chain that is one carbon shorter [89]. The same mechanism may also account for the observed C1 deletions, alternatively to a proposed Favorskii rearrangement, if the decarboxylation is oxidative [89], as explained below.

A single study has shown that the incorporation pattern of acetate units into YTX was highly similar as in BTX [65]. The polyether ladder backbone of YTX is assembled from intact acetate units with frequent interruptions by C1 deletions. In addition, several pendant methyl groups were derived from acetate methyl, apart from C-50, which was derived from SAM. Carbons C-1 and C-2 were neither labeled from acetate or SAM, and may be derived from a different extender unit, such as glycolate.

### 3.3. Linear polyether toxins

**Figure 4:**
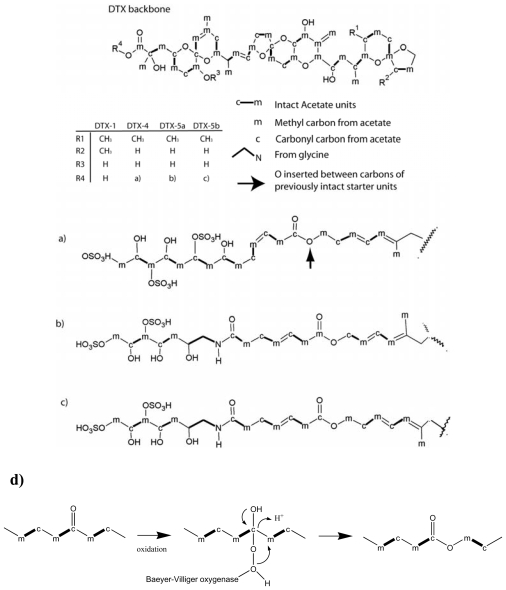
The linear polyethertoxin okadaic acid (OA) and analogs thereof. (**a**) side chain of dinophysis toxin-4, (**b**) side-chain of dinophysis toxin-5a, (**c**) side-chain of dinophysis toxin-5b. (**d**) Mechanism for oxygen insertion by a Baeyer-Villiger oxidation. m: acetate methyl, c: acetate carboxyl. Bold bonds between c and m indicate intact acetate units.

Linear polyethers include okadaic acid (OA) and dinophysistoxins (DTXs), which are produced in dinoflagellates of the genera *Dinophysis* and *Prorocentrum*. OA and DTX are the causative toxins for DSP, and inhibit protein phosphatase 2A and 1 [90]. DTX-1 is similar to OA, differing only by a methylated C-35 in DTX-1 (Figure 4). DTX-4, DTX-5a and -5b are extended analogs of OA that have sulfated, and poly-hydroxylated ester side-chains. In the case of DTX-5a and -5b, the side-chains also contain an amine (Figure 4).

Precursor incorporation studies have been performed on DTX-1 [91], DTX-5a and -5b [92], and OA [93–95]. These studies indicated that OA and the side-chains of DTXs are synthesized using glycolate as a starter unit, followed by consecutive addition of acetate units (Figure 4). As with dinoflagellate polyether ladder toxins, the pattern of consecutive intact acetate units is interrupted by C1-deletions, and pendant methyl side-chains are derived from acetate methyl carbons. The C1-deletions were proposed to be the result of Favorskii-like rearrangements [92]. Methyl- and methylene side chains derived from acetate methyl retained one or two deuteriums in labelling experiments with [2-^13^CD_3_] acetate. As a result, it was proposed that they are formed *via* an aldol condensation between a backbone carbonyl and acetate or malonate [95].

The side-chains of DTXs are linked to OA *via* an ester bond between the glycolate-derived hydroxy group, and the terminal carboxy group of OA (Figure 4). In addition, there is an ester bond in the mid section of the DTX side-chains. This ester oxygen is inserted between the carbonyl and methyl carbon of a previously intact acetate unit, which points to a Baeyer-Villiger oxidation (Figure 4) [92]. Precursor incorporation patterns into the side-chains were found to be identical in DTX-4 and DTX-5b, however DTX-5a is one carbon shorter, due to a C1 deletion. The precursor incorporation pattern thus indicated that the acetate C1 deletion precedes the Baeyer-Villiger oxygen insertion, and therefore C1 deletions must be an integral part of the polyketide synthesis process [95]. The terminal carbons of the side-chains in DTXs, and in OA also appear to be cleaved by a Baeyer-Villiger oxidation [95]. Finally, an intact glycine molecule is incorporated into the side-chain of DTX-5a/5b, where it replaces an acetate unit [92]. The replacement of acetate with glycine suggests that a PKS module has been exchanged with an NRPS module to create a hybrid NRPS/PKS synthetase [11]. Hybrid NRPS/PKS metabolites are known from bacteria, however amino acids are generally incorporated as starter or terminal units, and their mid-chain incorporation, such as in DTX-5a/5b is rare [92]. All hybrid NRPS/PKS investigated so far are modular enzymes [96], and it appears likely that OA and DTXs are synthesized by a modular hybrid NRPS/PKS system.

Mapping of the origins of oxygen atoms in OA and DTX has revealed a complex pattern, which suggested that OA synthesis may involve mechanisms that are active in the synthesis of polyketides from actinomycetes, as well as mechanisms that are unique to polyketide from dinoflagellates [94,97,98]. Whereas oxygens in monensin are derived from molecular oxygen, or carboxyl extender units [86], those of OA and DTX are derived from molecular oxygen, water, and carboxyl extender units. An unexpected observation was made, where the acetate-derived oxygens of OA were not labeled from H_2_^18^O unlike corresponding oxygens of salinomycin [98]. These results suggested a different route for the production of acetyl-/malonyl-CoA than from pyruvate or that ketone groups may become hydrated in nascent polyketide chains [98]. ^18^O-incorporation patterns proved, however, that the formation of rings C, D, E in OA proceeds *via* β-epoxidation of double bonds resulting from the elimination of the carboxylic acid-derived oxygen as in monensin biosynthesis [86,99].

### 3.4. Macrolides

**Figure 5:**
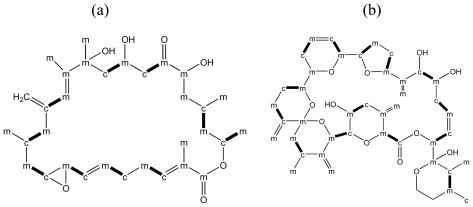

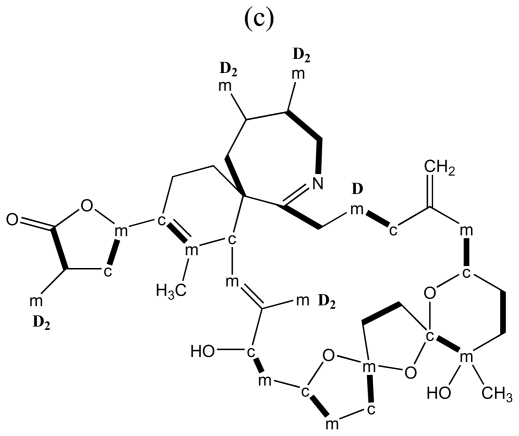
Incorporation patterns of stable isotope-labeled precursors into macrolides from dinoflagellates. (**a**) Amphidinolide B [81] (**b**) goniodimine A [82] (**c**) spirolde C [83]. Abbreviations used were: m acetate methyl, c acetate carbonyl, D deuterium (index indicates number of retained deuteriums). Bold bonds between c and m indicate intact acetate units.

Macrolides arise through the cyclization by internal esterification of linear polyethers, and a distinction between these two groups of metabolites is arbitrary. Macrolides from dinoflagellates include prorocentrolide, amphidinolides, pectenotoxin, goniodimin A, gymnodimine, spirolides, pectenotoxin, and carbemolides [6]. Biosynthetic studies have been performed on members of amphidinolide B, goniodomin A, and spirolides (Figure 5).

Amphidinolides form a large and heterogenous family of cytotoxic macrolides that are produced in symbiotic dinoflagellates of the genus *Amphdinium* [100]. They consist exclusively of acetate-derived carbons [11], which includes pendant methyl groups, and C1-deletions appear at least as frequently as in other polyketides from dinoflagellates (Figure 5). Careful measurements of intensity ratios of labeled *versus* unlabeled carbons supported that these deletions are due to a Farvorskii-type rearrangement or a similar process [81]. Interestingly, the positioning of acetate-derived methyl side-chains is unusual in amphidinolides. Most of the methyl groups are attached to the acetate-derived carbonyl carbons in the polyketide chain of amphidinolides, rather than to acetate-derived methyl carbons as in other dinoflagellate polyketides [11]. Another unusual but interesting feature is that although amphidinolide B and H share an identical carbon skeleton, they appear to be produced *via* two different pathways. In amphidinolide B, C-16 to C-19 are derived from two intact acetate units (cm-cm, where c stands for carbonyl, and m for methyl carbon of acetate), but the corresponding carbons of amphidinolide H showed an interrupted pattern (m-cm-c) [81,101]. For more information on the structures and biosynthetic origins of amphidinolides, the reader is referred to a detailed review by Kobayashi and Tsuda [100].

Goniodomin A is an antifungal macrocycle from the dinoflagellate *A. hiranoi* [82]. A biosynthetic study revealed incorporation of intact acetates that are frequently interrupted by C1-deletions (Figure 5). Two carbons (C-35 and C-36) were not labeled from acetate but were proposed to be derived from glycolate. Four pendant methylene and two pendent methyl groups were derived from acetate methyl, whereas one pendant methyl group was derived from SAM. The precursor incorporation pattern for goniodomin A is thus consistent with other dinoflagellate polyketides.

Spirolides are macrocyclic imine toxins, which also include pinnatoxin, gymnodimine, pteriatoxins, and spiro-prorocentrimine [83], and are produced by the dinoflagellate *A. ostenfeldii* [102]. The toxins are fast acting and target acetyl cholin receptors and calcium channels [83,103]. To date, thirteen isoforms of spirolides are known [104]. A biosynthetic study has shown that spirolide C was constructed from acetate units and an intact glycine in a fashion similar to other polyketides from dinoflagellates (Figure 5) [83]. The structure and biological activities of spirolides are reviewed by Guéret and Brimble in this issue [104].

### 3.5. Dinoflagellates produce polyethers by a distinct polyketide pathway

Stable isotope-labeled precursor feeding studies have firmly established the polyketide origin of polyether ladders, macrocycles, and linear polyethers from dinoflagellates [6]. However these toxins have several unusual structural and biosynthetic features in comparison to other polyketides. In general, the backbones of dinoflagellate polyketides are assembled from acetate, with minor additions of methionine methyl to form side-chains, and in some instances unusual starter units, such as glycolic acid, are used [6]. Single acetate methyl-derived carbons frequently interrupt the pattern of consecutive intact acetate units, and pendant methyl side-chains may be derived from acetate methyl carbons, rather than from conventional methyl donors, such as SAM. Furthermore, oxygens in dinoflagellate polyketides are of mixed origin from either molecular oxygen, water, or from the carbonyls of extender units [97,98]. The *trans*-fused polyether ladders are chemical structures that are entirely unique to dinoflagellates.

The suggestion that an incorporation of dicarboxylic acids from the citric acid cycle may account for the interrupted pattern of acetate incorporations was refuted, based on the incorporation of all acetate-derived carbons at an identical ratio [89]. An alternative explanation was that C1-deletions may be the result of a series of reactions, which occur after polyketide chain elongation. They involve the oxidation of an acetate-derived methyl carbon to yield a ketone, followed by a Favorskii rearrangement. This rearrangement yields a cyclopropanone and introduces a new connectivity between two acetate methyl carbons. Peroxidation of the carboxy carbon in the cyclopropanone breaks its connectivity to the adjacent acetate methyl carbon [95]. This series of reaction is consistent with the observed patterns of incorporated of acetate carbons, as well as their uniform ratios. In addition, Favorskii rearrangements have been implicated in the biosynthesis of polyether compounds in other organisms, however in these cases, the branched-out carbons are left as pendant carbonyl groups on the polyketide chain [89]. Their removal from the polyketide chain would require a decarboxylation [95].

Pendant single carbon groups that are derived from C1 acetate have not been observed in polyethers from dinoflagellates, casting some doubt regarding an involvement of Favorskii rearrangements in the C1 deletion process, and an alternative mechanism has been proposed [89]. In this scheme, carbon deletions may occur as an integral part of polyketide synthesis during the chain elongation process. The terminal β-ketone is reduced, and dehydrated to yield a double bond, as is conventional in polyketide and fatty acid biosynthesis. The double bond may then become epoxidated, followed by facile decarboxylation, or oxidative decarboxylation. The first case would yield a terminal aldehyde with an acetate methyl-derived carbon, and terminate polyketide extension. In the second case, the resulting carboxyl group containing an acetate methyl-derived carbon would facilitate further elongations. This scheme would involve polyketide synthase enzymes, which have additional integrated functional segments, such as epoxidase, and decarboxylase [89]. The presence of terminal aldehyde groups, which are built from acetate methyl derived carbons in several dinoflagellate polyether compounds, in addition to an absence of pendant single carbon groups that are derived from acetate carboxyl groups represent strong support for the latter scheme.

### 3.6. Biosynthesis of paralytic shellfish toxins

**Figure 6:**
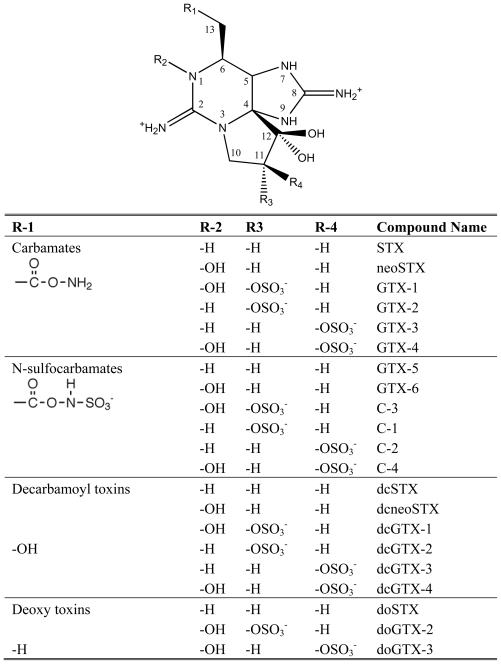
The molecular structure of saxitoxin and common analogs [112]. Abbreviations used are, STX: saxitoxin, GTX: gonyautoxin, C: C-toxin; prefixes mean: dc: decarbamoyl, do: deoxy.

Saxitoxin and its analogs cause paralytic shellfish poisoning (PSP), and are among the globally most pervasive and potent algal toxins. PSP is a severe form of seafood poisoning, which affects approximately 2000 people every year, and has a mortality rate of 15% [4]. The main action of saxitoxin analogs is a blockage of voltage-gated sodium channels [105] and they modulate the gating behaviour of calcium and potassium channels [106,107]. Gonyaulacoid dinoflagellates of the genus *Alexandrium,* and *Pyrodinium*, as well as a single gymnodinoid species, *Gymnodinium catenatum*, are producers of PSP toxins [4]. The chemical structure of saxitoxin is entirely different from polyether toxins described above. The backbone of PSP toxins consists of a tri-cyclic perhydropurine, which resembles purines of primary metabolism (Figure 6). Saxitoxin and its analogs are produced by a complex, and entirely unique biosynthesis pathway, in spite of the similarities to primary purines [108]. The building blocks are arginine, acetate, and methionine [108–110]. Arginine, minus its carboxylate group, the guanidino group of a second molecule of arginine, and an intact acetate unit form the tri-cyclic backbone. The methyl side-chain is derived from SAM, whereas the origin of the carbamoyl side-chain was uncertain but proposed to be derived from the guanidino group of a third molecule of arginine.

**Figure 7:**
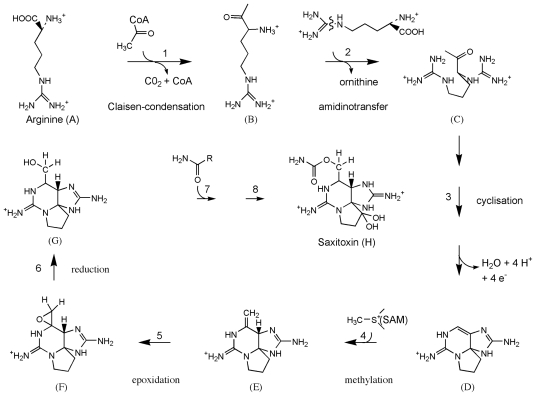
Biosynthesis pathway of saxitoxin as proposed by Shimizu *et al.* [111]. Hypothetical intermediate metabolites are labeled with letters in brackets. The reaction steps are: 1, Claisen-condensation between acetate and arginine; 2, amidino transfer from a second arginine to the α-amino group of intermediate B; 3, cyclization; 4, introduction of S-adenosyl methionine (SAM) methyl-derived side-chain, involving the loss of one methionine methyl hydride; 5, epoxidation of side-chain, leading to a 1,2-H shift; 6, opening of epoxide to an aldehyde followed by reduction of the aldehyde; 7 & 8, carbamoyl transfer and di-hydroxylation.

The incorporation pattern suggested a unique biosynthesis pathway that is initiated by a Claisen-condensation between acetate and arginine (Figure 7). The resulting intermediate would then be amidinated at its primary amino group and three heterocycles are formed. It was further proposed that the methyl side chain is introduced after cyclization, which is epoxidised, opened to an aldehyde, and dehydrogenated to a hydroxyl group. The incorporation of methionine methyl and its hydroxylation has been studied in detail. Only one methionine methyl-derived hydrogen is retained in saxitoxin, and a 1,2-hydride shift was observed between acetate-derived C-5 and C-6. Shimizu [111] proposed that this incorporation pattern resulted from an electrophilic attack of methionine methyl (see step 4 in Figure 7) on the double bond between C-5 and C-6, which would have formed during the preceding cyclization (see step 3 in Figure 7). Subsequently, the new methylene side chain would be epoxidated, followed by opening to an aldehyde and subsequent reduction to a hydroxyl (see steps 5 to 7 in Figure 7). This scheme should also lead to a 1,2-H shift between C-1 and C-5, however this was not been observed [111]. Finally, the saxitoxin precursor is di-hydroxylated at ring carbon C-12, and receives a carbamoyl group at its hydroxymethyl side-chain. Modifications at other positions may produce more than 30 saxitoxin derivatives (Figure 6).

Recently, the biosynthesis pathway for saxitoxin has been revised based on the genomic structure of the saxitoxin biosynthesis (*sxt*) gene cluster, and intermediate metabolite analysis [112,113]. The revised pathway contains essentially the same biochemical reaction, however their sequence is different. According to the revised pathway, saxitoxin biosynthesis is initiated by the SAM-dependent methylation of acetate to form propionate, which is followed by its Claisen-condensation to arginine. A subsequent reaction transfers a guanidino group to the free amino group and the first heterocycle is formed. The SAM-derived methyl group is desaturated, the resulting double-bond is epoxidated, and opened to an aldehyde with concomitant formation of two heterocycles. This scheme is consistent with the lacking 1,2-hydride shift between C-1 and C-5. Subsequent reactions then involve an *O*-carbamoylation of the hydroxymethyl side-chain from carbamoyl phosphateand two ring carbon hydroxylations to complete biosynthesis. At present, it is uncertain where the pathway branches off to produce modified analogs of saxitoxin.

Interestingly, dinoflagellates are not the only organisms capable of producing PSP toxins. Certain freshwater species of cyanobacteria also produce PSP toxins [114]. Precursor incorporation patterns, as well as the stereochemistry are identical in saxitoxin from dinoflagellates and cyanobacteria [111], which strongly suggests that these toxins are produced by homologous processes in both groups of organisms.

## 4. Phycotoxin Biosynthesis Genes

Success in identifying genes and enzymes that are involved in the biosynthesis of toxins by dinoflagellates has been limited thus far, in spite of considerable efforts. Hurdles to overcome are the enormous size and peculiarities of the dinoflagellate genome, difficulties in culturing these organisms, and the lack of methods for their genetic transformation. The chemical structures of polyketides from dinoflagellates suggest that they are produced by modular type I PKS enzymes in some cases with an involvement of a NRPS, for instance in the case of DTX-5a/5b and spirolides. A minimal PKS has an acyltransferase (AT) domain, a β-ketosynthase- (KS) domain, and an acyl carrier protein (ACP). The AT domain covalently transfers a specific carboxylic acid from acyl-CoA to the ACP, which is then condensed by the KS domain to another ACP-bound acyl chain. A PKS module may have optional β-ketoacyl reductase (KR), dehydrogenase (DH), and enoyl reductase (ER) domains, which reduce the β-ketone to an alcohol, dehydrate the alcohol, and saturate the resultant double bond, respectively. In analogy, a minimal NRPS provides an adenylation domain (A), which specifically activates an amino acid, a peptidyl carrier protein (PCP), and a condensation domain (C) that creates a peptide bond between two PCP-bound amino acids. Thioesterase (TE) domains may release, and cyclize the final enzyme products. Most studies have targeted conserved sequence regions of KS domains for the discovery of PKS genes in dinoflagellates.

### 4.1. An attempt to identify putative amphidinolide biosynthesis genes on the genomic level

Kubota *et al.* [115] screened genomic DNA from five amphidinolide producing and eight non-producing dinoflagellate strains by degenerate PCR for the presence of β-ketosynthase (KS) domains of type I PKS genes. Fragments of fourteen unique KS domains were detected. These sequences were exclusively present in amphidinolide producer strains, and a genomic fosmid DNA library was constructed from the amphidinolide producing strain *Amphidinium* sp. Y-42. Kubota *et al.* [115] detected a single clone out of a total of 100000 PCR-screened clones, which harbored a PKS-related sequences, and the entire fosmid insert (36.4 kb) was sequenced. The fosmid insert had six sequence regions, KS, AT, DH, KR, ACP, and TE that were related to type I PKS genes. Their genomic arrangement was unusual, as several frame-shifts occurred within and between catalytic domains. The protein-coding region was flanked on both sides by long stretches of non-coding sequence, and the mid section of the protein-coding region contained a 4 kb stretch of sequence that presumably represented an intron. Only approximately 15% of the 36.4 kb long fosmid insert consisted of protein-coding sequence. This sequence encoded putative catalytic functions for only a single elongation cycle of a 26-membered polyketide. If one would extrapolate based on these data, all genes required for the production of amphidinolide may occupy up to 500 kb of genomic DNA. Further, it would not be certain, whether they were present on the same locus, or distributed throughout the genome. This study exemplified the huge challenges associated with characterising biosynthesis genes in dinoflagellates on the genomic level. Other molecular genetic studies on polyketide biosynthesis in dinoflagellates used cDNA to avoid these kinds of difficulties. Unfortunately, sequences obtained in the study by Kubota *et al.* [115] were not deposited in GenBank, preventing further analysis.

### 4.2. Brevetoxins biosynthesis genes

Snyder *et al.* [116] screened for the presence of PKS β-ketosynthase genes by degenerate PCR, using cDNA from nine dinoflagellates strains representing seven species. A single type II PKS sequence was detected in the okadaic acid producing dinoflagellate *P. lima*, whereas type I PKS sequences were detected in seven strains, which represented six species (Table 1). Of the PKS-positive strains, only three strains were confirmed polyketide producers. The remaining strains have not been tested, but some of them were likely polyketide producer strains [116–118]. Only *A. operculatum* CCMP121 and *A. carterae* did not yield any PKS sequences. The unexpected presence of a PKS gene in *G. catenatum* may indicate that it produces an unknown polyketide.

Whereas the function of any of the obtained sequences was not investigated, some evidence was presented that supported an origin of the obtained sequences from the dinoflagellate genome. The PCR methods employed in the study by Snyder *et al.* [116] did not distinguish between sequences of dinoflagellate-, or associated bacteria origin. PKS genes were not expressed after light-deprivation and Southern hybridization with PKS-specific probes was positive for DNA isolated from dinoflagellate, but not for DNA isolated from associated bacteria [116]. Phylogenetic analysis did not reveal information regarding the evolutionary origin of dinoflagellate KS sequences. The authors suggested that this may be due to an ancient age of dinoflagellate KS, which may obscure their origin [116].

A follow-up study focused on PKS gene fragments obtained from brevetoxin-producing strains of *K. brevis* [119]. This study employed flow-cytometry-based sorting to separate bacteria from *K. brevis* cells, in addition to fluorescent *in situ* hybridization (FISH) to localize the presence of genes in *K. brevis* and associated bacteria. Two KS sequences (AT2-10L, AT2-15) were present in five brevetoxin producing strains of *K. brevis*, but absent from other dinoflagellates, their associated bacteria, and other protists examined. They therefore represented the most likely candidate brevetoxin biosynthesis genes. A third sequence (AT1-6L) was detected by PCR and sequencing in both the sorted algal, and bacterial fractions, as well as from two *Amphidinium* strains, which do not produce brevetoxin. FISH localized the AT2-10L gene to *K. brevis* cells, however the AT1-6L gene was present in both algal and a portion of bacterial cells [119]. The AS-1L sequence was no longer detectable in *K. brevis* in the follow-up study, and probably represented a bacteria-derived sequence [119]. Several other KS sequences (bac3, bac23, bac28, bac30), however, were obtained from the bacterial fraction of *K. brevis.* The follow-up study [119] thus conclusively determined the origin of KS sequences with regard to whether they were derived from the algae or associated bacteria.

Sequencing of approximately 25,000 ESTs from *K. brevis* uncovered six PKS related gene transcripts, with homology to ACP, KS, and KR [120]. Sequencing of the full-length transcripts revealed that they provided spliced leader sequences, the typical eukaryotic 3′-UTRs, and poly-A tails, which strongly suggested that they were encoded in the dinoflagellate genome. It also revealed that they encoded proteins, each with a single catalytic domain. The domain structure was thus more similar to type II PKS enzymes, whereas their sequences were closely related to type I PKS enzymes. An involvement of these sequences in brevetoxin biosynthesis was not demonstrated, however phylogenetic screening showed that two sequences occurred exclusively in brevetoxin producer strains, whereas the remaining four sequences were also present in non-brevetoxin producer strains. They may be involved in the biosynthesis of uncharacterized polyketides. Non-toxic dinoflagellates are known to possess PKS genes [121]. A distinct phylogeny of protist PKS sequences was supported by this and other studies [120,121].

### 4.3. Identification of a hybrid NRPS/PKS gene cluster from Karenia brevis

**Figure 8:**
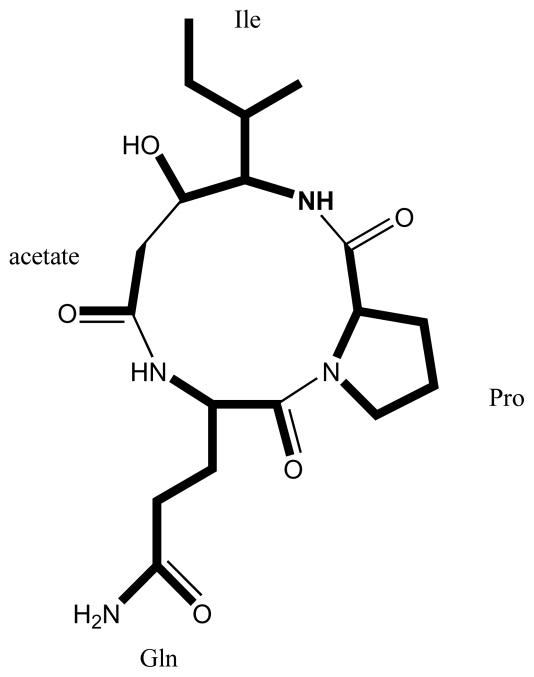
Hypothetical metabolite that was predicted from the genetic structure of a hybrid non-ribosomal peptide synthetase/polyketide synthase gene cluster, encoded in the chloroplast genome of *Karenia brevis* [88].

Genomic DNA from three strains of brevetoxin producing *K. brevis* was screened by degenerate PCR for the presence of PKS genes [88], and 18 unique KS sequences were detected. A fosmid library of genomic DNA from *K. brevis* 718 (Wilson) was constructed and seven out of 3840 screened clones contained PKS-related genes. The 16 kb long fosmid insert of one positive clone was sequenced. It encoded five open reading frames (ORFs). ORF1, 2 and 3 each encoded an NRPS with a condensation (C), adenylation (A) and peptidyl carrier protein (PCP) domain. ORF4 encoded a type I polyketide synthase with a KS, acyltransferase (AT), KR, and ACP domain, whereas the last ORF encoded a thioesterase (TE). A possible metabolic product related to this gene cluster was not predicted by this study. The structure of type I polyketides may be inferred from their biosynthetic genes, as modular type I PKS synthetases usually abide to the co-linearity rule [20]. Furthermore, substrates of A domains can be predicted based on their catalytic residues [122]. We used these methods to infer a hypothetical metabolite produced by the hybrid NRPS/PKS gene cluster identified by Lopez-Legentil *et al.* [88]. Catalytic site residues of the NRPS adenylation domains were identified using bioinformatics (http://202.54.226.229/~zeeshan/webpages/nrpsall.html), and are shown in Table 2. Bioinformatic inference suggested that the three NRPSs would synthesize a tripeptide, consisting of glutamine, proline and isoleucine. The hybrid PKS encoded by ORF 4 would then link the tripeptide to a terminal acetate unit, and reduce the isoleucine β-keto group to an alcohol. Finally, the TE would release the metabolite from the ACP, most likely with concomitant cyclization by condensation of the acetate carboxyl group to the glutamine α-amino group. The chemical structure of the hypothetical metabolite (Figure 8) does not resemble that of any metabolite isolated from a dinoflagellate so far; however, it has some resemblance to cyanobacterial peptolides.

Phylogenetic analysis did not place any of the eighteen new KS sequences among previously identified sequences from *K. brevis* or protists [116,119], however the majority grouped with KS sequences from cyanobacteria, including the identified hybrid PKS gene. Because of its affiliation with cyanobacterial PKS genes, chloroplast DNA from *K. brevis* was PCR screened with specific primers for the hybrid PKS with a positive result. The study concluded that the hybrid NRPS/PKS gene cluster was encoded in the chloroplast genome of *K. brevis* and that other identified KS sequences may have been acquired by *K. brevis* by horizontal gene transfer from an ancestral cyanobacterial endosymbiont.

An acquisition of secondary metabolite biosynthesis genes from endosymbionts, or their continuing presence in the chloroplast genome is intriguing. Localization of the hybrid NRPS/PKS gene cluster to the chloroplasts was based on organelle isolation, followed by DNA extraction, and PCR detection. Contamination of *K. brevis* culture and sub-cellular fractionations were excluded by 16S rDNA analysis, as well as by nested PCR targeting cyanobacterial nitrate transporter genes (López-Legentil, pers com.).

### 4.4. Polyketide synthase genes from DSP-toxin producing dinoflagellates

A single study has been performed to detect KS sequences of type I PKS in OA producing dinoflagellates of the genus *Prorocentrum* [123]. The study employed degenerate PCR with genomic DNA as a template, and uncovered 16 unique KS sequences from 280 cloned PCR products. Phylogenetic analysis placed none of these sequences among those from other protists, but grouped them separately, or together with bacterial KS sequences. The authors suggested that the sequences may have been derived from associated bacteria, rather than from the dinoflagellates. They refer furthermore to a possible link between bacteria and the production of DSP toxins [124].

### 4.5. Phylogenetic analysis of protist KS sequences

Type I PKS genes were previously only known from bacteria and fungi, however they have recently been found in alveolates [88,115,116,119,120,123], apicomplexan parasites, haptophytes, and chlorophytes [121,125]. Among protists, type I PKS genes are distributed in a patchy manner, and John *et al.* [121] have performed phylogenetic analyses to address questions regarding their origin. Protist type I PKS grouped separately from other known PKS genes and the study concluded that PKS genes in protists have not originated from recent horizontal gene transfer events. Two scenarios were presented for their likely evolution [121]. The last common ancestor of Plantae, Unikonta and Chromalveolata did possess type I PKS genes, which were lost in several descendant lineages, such as Rhodophyta, and higher plants. Alternatively, type I PKS genes were acquired independently in several eukaryotic lineages, for instance through gene duplication from FAS or lateral gene transfer events. An example for the latter scenario may be represented by the PKS CpPKS1 from *C. parvum*, which appears to have evolved through gene duplication from its fatty acid synthase, CpFAS1 [125].

The study by John *et al.* [121] did not include PKS genes from dinoflagellates; however, phylogenetic analyses performed by Snyder *et al.* [119], Lopez-Legentil *et al.* [88], and [123] included dinoflagellate and apicomplexan sequences. The study by Snyder *et al.* [119] was the only study, which demonstrated the source of the sequences and from which these sequences were publicly available. In this study, sequences derived from *K. brevis* grouped with other alveolate protist sequences and were separated them from non-protist sequences. Sequences obtained from bacteria that were associated with *K. brevis* grouped with other bacterial sequences. Two notable exceptions occurred. AT1-6L, which was present in both bacterial and algal cells, as well as bac30, which was only present in bacteria, grouped with the protist sequences. The grouping of protist sequences suggests a common origin of alveolate PKS genes. The presence of an identical sequence, AT1-6L, in both bacterial and *K. brevis* cells and its phylogenetic placement within the protist clade together with another bacterial sequence (bac30) is intriguing. It may indicate that horizontal gene transfer may have occurred from dinoflagellates to bacteria.

The hybrid NRPS/PKS sequences obtained by Lopez-Legentil [88] grouped together with sequences from cyanobacteria and *in silico* inference of the chemical structure of its associate metabolite product suggested resemblance to peptolides from cyanobacteria. Whereas there is little doubt regarding the phylogenetic affiliation of this gene cluster with cyanobacteria, its source will have to be verified. Finally, PKS sequences from OA producing *Prorocentrum* sp. grouped mainly with other bacteria [123], however most, if not all of these sequences were probably derived from bacteria that were associated with the algal cultures.

### 4.6. Biosynthesis genes and enzymes of paralytic shellfish toxins

Several approaches have been used to identify biosynthesis genes and enzymes of saxitoxin and its analogs in dinoflagellates. They included the use of differential display [126], purification of enzymes in conjunction with activity assays [127,128], and shotgun EST sequencing [129].

It has been observed that the production of PSP toxins was linked to the G(1) phase of the growth cycle in *A. fundyense* [130,131]. This observation was the basis for a study, which attempted to identify genes that were upregulated during G(1) phase by differential display, and thereby hoped to include PSP toxin biosynthesis genes [126]. The study identified 21 differentially regulated genes that had functions in the cell cylce, in addition to three genes, which encoded S-adenosylhomocysteine hydrolase (Sahh), a methionine aminopeptidase (Map), and a histone-like protein (HAf). A possible function of the latter three enzymes in PSP toxin was not apparent. Map and HAf have likely functions in protein biosynthesis, and chromosome organization, respectively. Sahh may have a key role in the salvage pathway for the bi-product S-adenosylhomocysteine that is generated in methylation reactions involving SAM-dependent methyltransferase. Because PSP toxin biosynthesis involves a SAM-dependent methyltransferase, an indirect relationship between Sahh and PSP toxin biosynthesis may exist. However, Sahh was downregulated during G(1) phase, and the nature of a possible link was not clear. The study by Taroncher-Oldenburg [126] only accounted for a possible transcriptional regulation of PSP toxin biosynthesis, however other studies suggest that PSP toxin biosynthesis enzymes are long-lived enzymes with a slow turn-over and may be regulated by post-translational mechanisms [112]. Dinoflagellates are known to provide cell-signaling pathways that are regulated through protein phosphorylation cascades, such as those found in other eukaryotic organisms [132–135]. Post-translational modifications of PSP toxin biosynthesis enzymes is therefore one of the likely mechanism, which may regulate toxin production.

Alternatively to searching for biosynthesis genes, several studies attempted to purify and identify enzymes, which are involved in the production of PSP toxins. The activities of two 3′-phospho-S-adeonsine 5′-phosphosulfate (PAPS) dependent sulfotransferases, N-ST and O-ST, was detected in extracts from *G. catenatum, A. catenella* [127,128,136], and *A. tamarense* [137]. N-ST sulfated the carbamoyl-amine of STX, and GTX-2/3 to produce GTX-5 and C1+2, respectively, however it did not sulfate the carbamoyl-amine of N-1 hydroxylated toxins, such as neoSTX, and GTX-1/4 [127]. O-ST sulfated the hydroxyl group of 11-hydroxySTX, which is thought to be the intermediate between STX and O-22 sulfated STX analogs [128]. Both enzymes had high substrate specificity, as there was no overlapping activity between N-1, and O-22 sulfation. Furthermore, N-ST and O-St could not sulfated common substrates of other sulfotransferases, such as p-nitrophenol, naphtylamine, L-tyrosine methyl ester, L-tyramine, dopamine, epinephrine, and estron. NS-T and O-ST had properties that were unusual among sulfotransferases. Monometic N-ST (60 kDa) and O-ST (67 kDa) were approximately twice the size of other sulfotransferases, which are between 30 and 36 kDa [138]. Comparison of PSP-ST between different species revealed great variation in properties, such as cation requirement, and optimum temperature [136,137]. N-ST and O-ST had lower specific activities (317 and 1691 pmol/mg per min) than most other sulfotransferases [139,140]. N-ST from *G. catenatum* had a requirement for bivalent cations, such as Mg^2+^ or Co^2+^ [127]. In addition, the optimum temperature of N-ST from *A. catenella* was 15 °C, whereas that from *G. catenatum* was 25 °C. The high specificities of O-ST and N-ST indicated that they were in fact biosynthesis enzymes for PSP toxin analogs. Unfortunately, the study failed to purify sufficient amounts of protein required for amino acid sequencing [127,128]. The enzyme activities of N-ST and O-ST indicate the order of reactions, such that STX may either be sulfated by N-ST to produce GTX-5 or hydroxylated to produce 11-hydroxySTX, which may then be sulfated by O-ST to form GTX-2/3. N-ST may then also sulfate the carbamoyl-amine of GTX-2/3 to form C-1/2 [128].

Shotgun sequencing of normalized EST libraries has been performed on *A tamarense* (10885 ESTs) [129], and *A. catenella* (9848 ESTs) [141]. However putative PSP biosynthesis genes were not identified in these studies.

**Figure 9:**
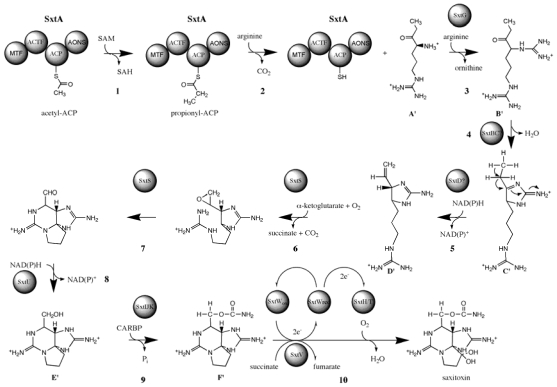
Revised biosynthesis pathway of saxitoxin [113]. Labeled spheres represent enzymes, or catalytic domains encoded by the *sxt* genes. Abbreviations used were, MTF: methyltransferase, ACTF: GCN-5 related acetyl transferase, ACP: acyl carrier protein, AONS: 8-amino-7-oxononanoate synthase-related domain, SAM: S-adenosyl methionine, SAH: S-adenosyl homocysteine.

In addition to marine dinoflagellates, certain freshwater species of cyanobacteria also produce PSP toxins [114,142]. A distribution of a complex and unique metabolic pathway between organisms from two kingdoms of life, which in addition inhabit ecologically distinct environments, is highly unusual. Suggested reasons for this have been that that saxitoxin biosynthesis may have a polyphyletic origin [143], that it may have been acquired through symbiotic bacteria [144,145], or that the genes may have spread through horizontal gene transfer [146]. The fact that the biosynthesis pathway for PSP toxins, as well as their stereo-chemistry is identical in dinoflagellates and cyanobacteria is a strong indication that these toxins are produced by homologous enzymes in both groups of organisms. Recently, a series of studies led to the identification of the PSP toxin biosynthesis gene cluster (*sxt*) in cyanobacteria [112,113,147,148]. The *sxt* gene cluster from *Cylindrospermopsis raciborskii* T3 spans approximately 35 kbp, and encodes 31 open reading frames [113]. Of these, 17 genes were predicted to encode enzymes that are directly involved in PSP-toxin biosynthesis. A novel polyketide synthase, SxtA, initiates PSP toxin biosynthesis. This enzyme has four catalytic domains with predicted activities of SAM-dependent methyltransferase (MT), GCN-5 related N-acetyltransferase (GNAT), acyl carrier protein (ACP), and class II aminotransferase (AT), and it is though to catalyse the loading of ACP with acetate, its subsequent methylation, and Claisen-condensation of the resulting product with arginine (Figure 9). Subsequently, the amidinotransferase, SxtG, transfers a guanidino group from arginine to the first intermediate. The first heterocycle is then most likely formed by the cytidine deaminase, SxtB, and the sterole-desaturase related enzyme, SxtD may introduce a double bond between the two most distal carbons. An α-ketoglutarate dependent dioxygenase, SxtS, was then predicted to epoxidate the new double bond, which is opened to an aldehyde with concomitant formation of two heterocycles. The side-chain aldehyde may then be reduced to an alcohol by the short-chain dehydrogenase, SxtU, and a carbamoyl group is then transferred from carbamoyl phosphate to the new hydroxyl group. Finally, two highly similar phenylpropionate dioxygenase related enzymes, SxtT and SxtH, might consecutively hydroxylate C-12. Terminal oxygenases, such as SxtT and SxtH, require regeneration after each catalytic cycle by an oxygenase reductase. The latter function may be performed by succinate dehydrogenase (SxtV), and ferredoxin (SxtW), using succinate as an electron donor. In this pathway, succinate may be supplied by SxtS, which converts α-ketoglutarate to succinate during the epoxidation reaction.

In addition to biosynthesis enzymes, the *sxt* cluster encoded a range of genes with other and unknown functions. Two enzymes were identified, which may be involved in the conversion of STX analogs. SxtN was predicted to act as a sulfotransferase, which converts STX and GTX-2/3 into GTX-5 and C-1/2, respectively. Studies on dinoflagellates indicated that PAPS is the sulfate donor for N-ST and O-ST, as described above, and the *sxt* gene cluster encoded its own adenylyl sulfate kinase, SxtO, which catalyses the formation of PAPS-precursor. The cephalosporin hydroxylase-like enzyme, SxtX, was only detected in strains that are able to produce N-1 hydroxylated analogs of STX and its function was correspondingly predicted to be a N-1 hydroxylase. Further enzymes encoded by the *sxt* gene cluster had various other predicted functions, including binding and transport of toxins, transposition of genes, transcriptional regulation, and unknown functions.

An involvement of *sxt* genes in saxitoxin biosynthesis was predicted by *in silico* inference and supported by several lines of evidence. Phylogenetic screening revealed that *sxt* genes were exclusively present in PSP-toxic strains of cyanobacteria. In addition, the genetic structure of *sxt* genes suggested a different sequence of biosynthetic reactions than previously predicted [108,110], and correspondingly, a different structure of biosynthetic intermediates. Several intermediate metabolites predicted from the structure of *sxt* genes were detected by LC-MS and their fragmentation spectra verified the assumed molecular structures [113]. Finally, the function of the O-carbamoyltransferase, SxtI, was ascertained. It was predicted to be responsible for the formation of the side-chain *O*-carbamoyl-group of STX analogs. Phylogenetic PCR-screening and sequencing of *sxtI* revealed that *L. wollei* was the only PSP-toxic strain, which had a non-functional *sxtI* gene, as it had two deletions in its active site, and lacked a large C-terminal fragment, which included part of the catalytic site [147]. This strain was shown by HPLC and NMR to be incapable of producing carbamoylated analogs of STX [149].

A translated blast search of the EST libraries from *A. tamarense* and *A. catenella* did not reveal sequences with significant homology to any of the *sxt* genes. A single EST from *A. tamarense* had some similarity to a region of *sxtA* from *C. raciborskii* T3 [150], but its involvement in saxitoxin biosynthesis is uncertain. The failure to detect *sxt*-like sequences may be the result of a low coverage of the EST library (approximately 60%) in addition to the short length of obtained sequences [129].

## 5. A Search for *sxt* Genes in the Oceans

As discussed above, neurotoxic saxitoxins are synthesized by selected species of both freshwater cyanobacteria and marine dinoflagellates. The synthesis of the same toxins through likely similar pathways by such evolutionary distant organisms, which live in different habitats, is astonishing.

The evolutionary history, which led to this unusual distribution of saxitoxin biosynthesis genes, remains to be elucidated. It is unclear, if the pathway arose independently in both lineages, if it was present in an ancient ancestor of cyanobacteria and dinoflagellates, or if it was horizontally transferred at a later stage. Alternatively, saxitoxins might not be produced by dinoflagellates themselves, but by symbiotic bacteria, a hypothesis that has been investigated by several studies; the results, however, are contradictory [151–153].

The Global Ocean Sampling (GOS) Expedition is an enormous effort to provide a comprehensive genomic survey of microbial life in the world’s oceans. Scientists of the J. Craig Venter Institute (JCVI) sail all oceans and simultaneously take plankton samples every ~200 km before, these are subsequently sequenced by environmental shotgun sequencing technologies [154–156]. The sequence data is made publicly available through the CAMERA website [157]. If the organisms that synthesize saxitoxin in the marine environment, whether or not these are dinoflagellates or bacteria, were present at the GOS sampling sites, sequences of these marine saxitoxin genes are likely to be present in this enormous dataset. If, in addition, these genes have some sequence similarity to the saxitoxin genes identified from freshwater cyanobacteria, mining the CAMERA database may help to identify these genes in the oceans.

Thus, we BLAST searched (tBLASTn) the GOS dataset for genes and gene fragments having sequence similarities to the saxitoxin genes described from freshwater cyanobacteria (for exact methods see supplementary material). In addition to the GOS dataset, we included all other metagenomic datasets and prokaryotic genome datasets available through CAMERA, as well as all proteins included in the NCBI non-redundant protein database. The reasoning behind including these additional databases in our analyses was to investigate whether or not genes with a high similarity to the cyanobacterial saxitoxin genes are present in any of these datasets.

**Figure 10:**
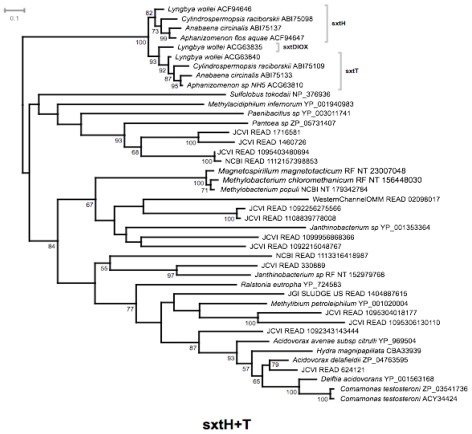
Phylogeny of phenylpropionate dioxygenase proteins SxtH, and SxtT. The tree was constructed from the closest hits from the CAMERA “all metagonomic sequence reads”, “all metagonomic 454 reads”, “all prokaryotic genomes”, and NCBInr databases. The tree was reconstructed with Maximum Likelihood (RAxML). Numbers on the internal nodes represent bootstrap values (>50%).

The results show that saxitoxin genes from cyanobacteria always cluster together with full or high statistical bootstrap support (Figure 10, and supplementary Figures 1 to 4). Our analyses were focused on the essential enzymes SxtA, SxtG, SxtI, SxtH and SxtT, but the same pattern also emerged from preliminary analyses of the conserved enzymes SxtB, C, D, E, M, N, P, Q, R, S, P and SxtU (data not shown). The two dioxygenases, SxtH and SxtT, placed together as a well-supported monophyletic clade, which was subdivided into two highly supported sister-clades (Figure 10). The sister-clade, which contained SxtT, also contained the SxtDIOX sequence from *Lyngbya wollei* (Figure 10). The close phylogenetic relationship of these two genes points to a duplication event. In the case of *L. wollei*, gene duplication might have happened twice. Sequences from the metagenomic datasets, the prokaryotic genomes, or from the NCBI protein database were not included in any of the clusters saxitoxin genes. We could thus not find any evidence for the presence of saxitoxin genes with sequence homology to those from freshwater cyanobacteria.

This finding might be due to the absence of such genes in the oceans. It is possible that the saxitoxin genes from marine saxitoxin producers are so different in sequence from the genes of freshwater cyanobacteria that they were not detected in the present analysis. This could be interpreted as contradictory of a recent HGT event between bacteria and dinoflagellates. Alternatively, no saxitoxin-producing organisms were present in the samples as a result of the oceanic sampling method. Many of the saxitoxin-producing dinoflagellates are larger than 20 μm and would have been excluded from the metagenomic samples by the 20 μm filter employed by the GOS sampling team [155]. However some species, such as *A. minutum* might have passed through this pre-filtering step. Mining the dataset for sequences with similarity to rRNA genes from the genera *Gymnodinium* and *Alexandrium* and analyzing the results with the metagenome analysis software MEGAN [158], indicated that DNA from these two genera was present in at least some the samples (supplementary Figures 4 and 5). Whether the DNA stemmed from toxin-producing strains remains unclear. However, bacteria would have easily passed through the pre-filter. Our results may thus indicate that marine bacteria do not harbor genes with a high sequence similarity to saxitoxin genes from freshwater cyanobacteria.

Any firm conclusions are difficult to make from these investigations. Saxitoxin data were not available for any of the datasets included here, and it may be possible that saxitoxin-producing organisms were not present when the GOS samples were taken. Thus, further studies are required to elucidate the evolutionary history of saxitoxin biosynthesis.

## 6. Possible Applications and Future Outlook

Polyether compounds from dinoflagellates have complex structures that are produced by polyketide pathways, which are distinct from those of other organisms. Similar types of mechanisms that appear to be unique to dinoflagellates are active in all examples of polyether metabolites from dinoflagellates that have been investigated. The unique biochemistry, and the phylogenetic clustering of certain dinoflagellate PKS genes with those from protists [119,121] may suggest that these polyketide pathways have originated and evolved in ancestral protists, and may not have been acquired through recent HGTs from bacteria or endosymbionts. On the other hand, several other dinoflagellate PKS genes were more closely related to bacterial PKS genes [119], and a hybrid NRPS/PKS gene cluster was located to the dinoflagellate chloroplast [88]. Taken together, these observations suggest that dinoflagellates may provide polyketide pathways that are intrinsic, as well as those that have been acquired by HGT and EGT. It is essential, however, to determine the function of the PKS genes that have been obtained from dinoflagellates, in order to conclusively verify such claims.

Research into the molecular genetics of polyketide synthesis in dinoflagellates has only just started. Many hurdles have to be overcome before the function of PKS genes can be addressed. As opposed to prokaryotes, where genes of biosynthetic pathways are typically clustered [159], there is no indication that this may also be the case in dinoflagellates. Studies at the genomic level have shown, on the other hand, that large distances of non-coding regions exist between protein-coding regions, and that the structure of modular PKSs, which are multi-domain enzymes in other organisms, can be encoded as single-domain enzymes in dinoflagellates. The excessive sizes and peculiarities of dinoflagellate genomes make the identification and characterization of biosynthesis pathways on the genetic level challenging, at the very least. Employing high throughput sequencing of toxin-producing dinoflagellates at the mRNA (cDNA) level is likely to be rewarding. For example, using the 454 long read technology on cDNA from dinoflagellate species producing toxins should provide a wealth of useful information. However, new strategies have to be developed for the functional assignment of genes. Few studies have attempted the genetic transformation of microalgae [160]. This has been achieved for some species, including the dinoflagellates *Amphidinium* and *Symbiodinium* [161], and would represent a valuable tool in conjunction with phenotypic screening, for the characterization of biosynthetic pathways in dinoflagellates. Alternatively, dinoflagellate genes may be heterologously expressed to determine their function. The latter approach would require substantial previous knowledge regarding the enzymes necessary for the pathway in question.

The benefits of succeeding in the molecular genetic characterization of polyketides from dinoflagellates will be many. They include the provision of tools for the direct detection of toxin genes in environmental samples. The complex and unique structures, as well as their novel biochemistries make such studies highly relevant from a biotechnological point of view. Such knowledge may provide valuable tools to add to the catalytic collective that is available for combinatorial biosynthesis of future medicines.

## Figures and Tables

**Table 1:** Dinoflagellate strains PCR-screened for the presence of PKS genes [116].

Species	Strain	type I PKS	type II PKS	polyketide
*Prorocentrum lima*		+	+	okadaic acid
*Prorocentrum hoffmanianum*		+	−	okadaic acid
*Karenia brevis*	CCMP718	+	−	brevetoxin
*Symbiodinium* sp.	CCMP831	+	−	ND
*Amphidinium operculatum*	CCMP1342	+	ND	ND
*Amphidinium operculatum*	CCMP120	+	ND	ND
*Amphidinium operculatum*	CCMP121	−	ND	ND
*Amphidinium carterae*	*CCMP1314*	−	ND	ND
*Gymnodinium catenatum*		+	ND	ND

+ present; − absent; ND not determined.

**Table 2:** Catalytic site residues of adenylation domains identified by Lopez-Legentil *et al.* [88] and those with known substrate-specificities.

Name	Catalytic Residues	Known Substrates	Predicted Substrates
tyroc003	DAWQFGLIDK	GLN	
McnA	DAWQTGLIDK	GLN	
NRPS-1	DAWQFGLIDK		GLN

Syp-M2	DVQYIAHVTK	PRO	
ituri002	DVQFIAHVXK	PRO	
NRPS-2	DVQFIAXXXK		PRO

bacit001	DGFFLGVVYK	ILE	
NRPS-3	DAFFLGVTYK		ILE
